# Exploring the Bacteriome and Resistome of Humans and Food-Producing Animals in Brazil

**DOI:** 10.1128/spectrum.00565-22

**Published:** 2022-08-22

**Authors:** Fabíola Marques de Carvalho, Tiago Barcelos Valiatti, Fernanda Fernandes Santos, Alessandro Conrado de Oliveira Silveira, Ana Paula C. Guimarães, Alexandra Lehmkuhl Gerber, Cintya de Oliveira Souza, Dandara Cassu Corsi, Danielle Murici Brasiliense, Débora de Souza Collares Maia Castelo-Branco, Eleine Kuroki Anzai, Francisco Ozório Bessa-Neto, Glaucia Morgana de Melo Guedes, Gleyce Hellen de Almeida de Souza, Leandro Nascimento Lemos, Lúcio Fábio Caldas Ferraz, Márcia de Nazaré Miranda Bahia, Márcia Soares Mattos Vaz, Ramon Giovani Brandão da Silva, Ruanita Veiga, Simone Simionatto, Walter Aparecido Pimentel Monteiro, William Alencar de Oliveira Lima, Carlos Roberto Veiga Kiffer, Antonio Carlos Campos Pignatari, Rodrigo Cayô, Ana Tereza Ribeiro de Vasconcelos, Ana Cristina Gales

**Affiliations:** a Bioinformatics Laboratory, National Laboratory of Scientific Computing (LNCC), Rio de Janeiro, Rio de Janeiro, Brazil; b Universidade Federal de São Paulo (UNIFESP), Laboratório Alerta, Division of Infectious Diseases, Department of Internal Medicine, Escola Paulista de Medicina (EPM), São Paulo, São Paulo, Brazil; c Regional University of Blumenau (FURB), Blumenau, Santa Catarina, Brazil; d Seção de Bacteriologia e Micologia, Instituto Evandro Chagas (IEC), Secretaria de Vigilância em Saúde (SVS), Ministério da Saúde, Ananindeua, Pará, Brazil; e Postgraduate Program in Medical Microbiology, Group of Applied Medical Microbiology, Federal University of Ceará (UFC), Fortaleza, Ceará, Brazil; f Universidade Federal de São Paulo (UNIFESP), Laboratório de Imunologia e Bacteriologia (LIB), Setor de Biologia Molecular, Microbiologia e Imunologia, Departamento de Ciências Biológicas (DCB), Instituto de Ciências Ambientais, Químicas e Farmacêuticas (ICAQF), Diadema, São Paulo, Brazil; g Universidade Federal da Grande Dourados (UFGD), Laboratório de Pesquisa em Ciências da Saúde, Dourados, Mato Grosso do Sul, Brazil; h Laboratory of Molecular Biology of Microorganisms, University São Francisco (USF), Bragança Paulista, São Paulo, Brazil; i Universidade Federal de São Paulo (UNIFESP), Laboratório Especial de Microbiologia Clínica (LEMC), Division of Infectious Diseases, Department of Internal Medicine, Escola Paulista de Medicina (EPM), São Paulo, São Paulo, Brazil; University of West London

**Keywords:** One Health, drug resistance, surveillance, metagenomics, antimicrobial resistance genes, bacterial communities

## Abstract

The epidemiology of antimicrobial resistance (AMR) is complex, with multiple interfaces (human-animal-environment). In this context, One Health surveillance is essential for understanding the distribution of microorganisms and antimicrobial resistance genes (ARGs). This report describes a multicentric study undertaken to evaluate the bacterial communities and resistomes of food-producing animals (cattle, poultry, and swine) and healthy humans sampled simultaneously from five Brazilian regions. Metagenomic analysis showed that a total of 21,029 unique species were identified in 107 rectal swabs collected from distinct hosts, the highest numbers of which belonged to the domain *Bacteria*, mainly *Ruminiclostridium* spp. and *Bacteroides* spp., and the order *Enterobacterales*. We detected 405 ARGs for 12 distinct antimicrobial classes. Genes encoding antibiotic-modifying enzymes were the most frequent, followed by genes related to target alteration and efflux systems. Interestingly, carbapenemase-encoding genes such as *bla*_AIM-1_, *bla*_CAM-1_, *bla*_GIM-2_, and *bla*_HMB-1_ were identified in distinct hosts. Our results revealed that, in general, the bacterial communities from humans were present in isolated clusters, except for the Northeastern region, where an overlap of the bacterial species from humans and food-producing animals was observed. Additionally, a large resistome was observed among all analyzed hosts, with emphasis on the presence of carbapenemase-encoding genes not previously reported in Latin America.

**IMPORTANCE** Humans and food production animals have been reported to be important reservoirs of antimicrobial resistance (AMR) genes (ARGs). The frequency of these multidrug-resistant (MDR) bacteria tends to be higher in low- and middle-income countries (LMICs), due mainly to a lack of public health policies. Although studies on AMR in humans or animals have been carried out in Brazil, this is the first multicenter study that simultaneously collected rectal swabs from humans and food-producing animals for metagenomics. Our results indicate high microbial diversity among all analyzed hosts, and several ARGs for different antimicrobial classes were also found. As far as we know, we have detected for the first time ARGs encoding carbapenemases, such as *bla*_AIM-1_, *bla*_CAM-1_, *bla*_GIM-2_, and *bla*_HMB-1_, in Latin America. Thus, our results support the importance of metagenomics as a tool to track the colonization of food-producing animals and humans by antimicrobial-resistant bacteria. In addition, a network surveillance system called GUARANI, created for this study, is ready to be expanded and to collect additional data.

## INTRODUCTION

The consequences of commensal or unsuspected microorganisms for the health of both animals and humans are often underestimated. It is estimated that 58% of microorganisms known to be pathogenic to humans can be transmitted by animals (1). Furthermore, 73% of pathogenic species reported to be emerging or reemerging have zoonotic origins (1). To achieve a rapid response to mitigate disease, it is essential to investigate entire microbial communities, including both pathogenic and nonpathogenic microorganisms. Microbiota shared among humans and animals must be considered under a One Health approach, as continuous environmental changes and close contact with animals can impact human health (2).

Antimicrobial resistance (AMR) is considered a global public health problem (3, 4). According to the most recent CDC report, more than 2.8 million antibiotic-resistant infections occur in the United States each year, resulting in more than 35,000 deaths (5). A similar number of deaths (33,110) attributed to antimicrobial-resistant infections has been estimated in European countries using EARS-Net data collected in 2015 (6). Although a large proportion of AMR infections are health care-associated infections (7), several studies have documented the emergence and spread of antimicrobial-resistant pathogens in community settings (8–10), especially those pathogens causing foodborne and urinary tract infections (7). Overdevest et al. (11) showed that clones of Escherichia coli isolated from humans and poultry meat in the Netherlands shared the same extended-spectrum-β-lactamase (ESBL)-encoding gene. Additionally, Leverstein-van Hall et al. (12) demonstrated the presence of an IncI1 plasmid that carried *bla*_CTX-M-1_ or *bla*_TEM-52_ among E. coli isolates recovered from poultry and bloodstream and urinary tract infections.

The selective pressure exerted mainly by the massive use of antimicrobials has had an unprecedented impact on the spread of antimicrobial-resistant pathogens (13). According to the World Organization for Animal Health (OIE), 41% of 146 countries that use antimicrobials in livestock for prophylaxis or treatment allow their use as growth promoters (14). Making the situation even worse, most of these antimicrobials show broad-spectrum activity and are also prescribed for humans (14). According to a previous study by Van Boeckel et al. (15), Brazil ranks third in the consumption of antimicrobials in food animal production. Although those authors reported that China and India represent the largest sources of antimicrobial resistance genes (ARGs) in animals and food products from developing countries, the scarcity of data from South America makes it difficult to estimate the actual occurrence of ARGs in this geographic region (16). Humans and animals share the same environment, are epidemiologically related, and are directly involved in ARG acquisition and dissemination (4, 17). In this context, the need to detect ARGs in distinct ecological niches justifies the implementation of surveillance based on the One Health approach (18, 19). In addition, ARGs can be easily transferred from the environment to human-pathogenic bacteria and vice versa due to horizontal gene transfer (13). Strategies for controlling AMR dissemination have been widely discussed due to the direct and indirect impacts on global public health and the global economy (20). Metagenomic tools have been widely used in surveillance projects conducted in high-income countries to verify the compositions of different microbiomes as well as the occurrence of ARGs, providing more accurate results than conventional culture methods (21–24).

Brazil is the fifth largest country by area (3.2 million mi^2^) and represents 47% of the South American continent. It is also the sixth most populous country in the world (212,6 million inhabitants), the largest exporter of beef (2,359 million tons) and poultry (3,875 million tons), and the fourth largest exporter of pork (1,178 million tons) according to a 2020 report (25), with Asian countries being the main importers of these animal products (26). As part of a One Health-based surveillance study, we characterized the fecal metagenomes of food-producing animals (poultry, cattle, and swine) and humans from specimens collected in the same time frame from all five Brazilian geographic regions as well as the resistomes encountered as part of the GUARANI (One Health Brazilian Group) network. To the best of our knowledge, this is the first study of this kind conducted in South America.

## RESULTS

### Diversity and host microbial characterization.

**FIG 1:**
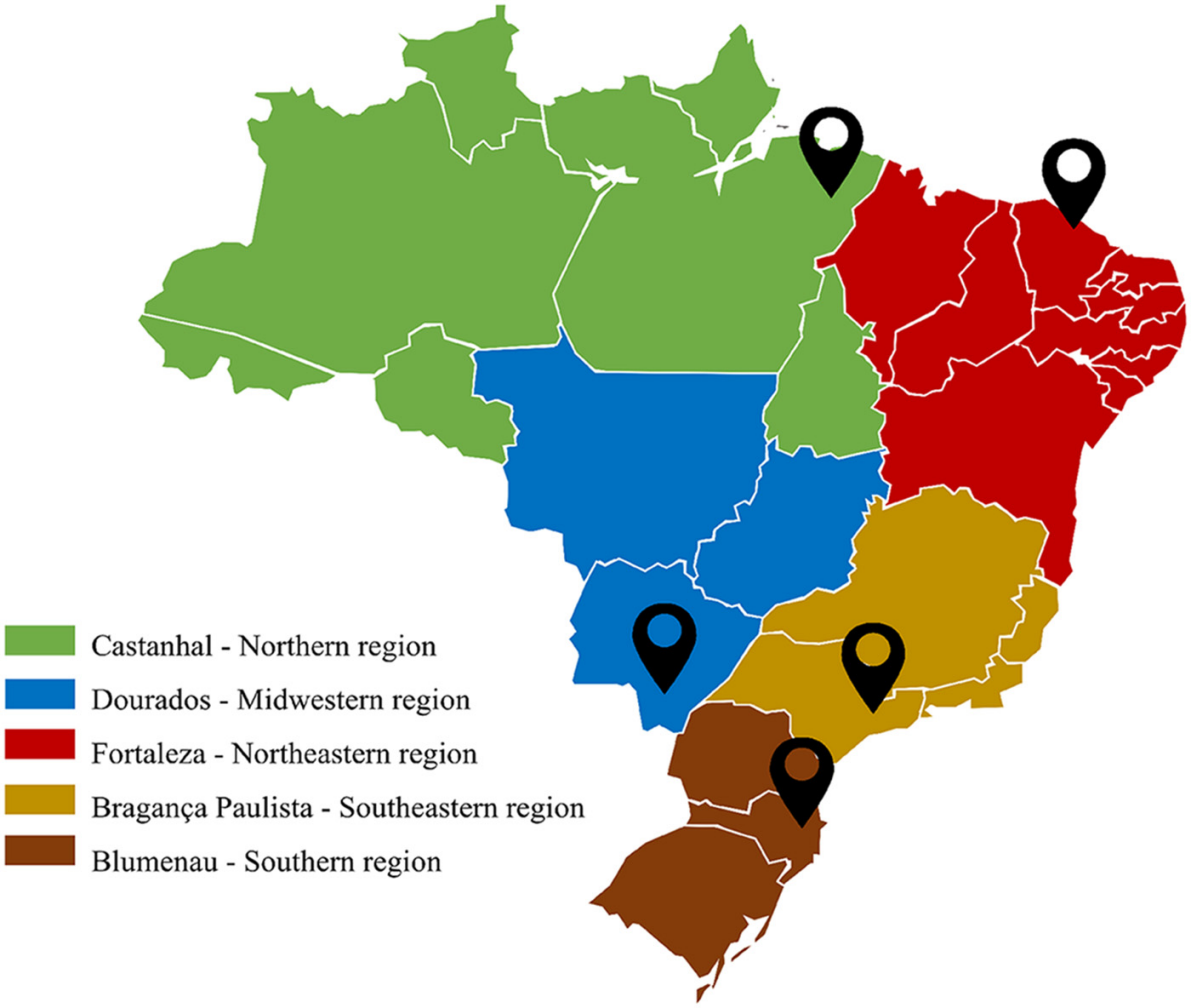
Map of Brazil showing the geographic locations of the five participating centers.

The microbial compositions in 107 samples collected from poultry (*n* = 30), cattle (*n* = 30), swine (*n* = 15), and humans (*n* = 32) from all five Brazilian geographic regions were determined (Fig. 1). Each data set had three replicates, totaling 321 individual samplings. The average number of trimmed reads by sample varied from 1,474,376 to 49,909,522, leading to a total of 1.62 billion bp. Host-derived reads over all samples were poorly represented, with a frequency of <0.01% in the majority of samples. The median number of *N*_50_ contigs was 4,160, and that of coding sequences of genes was 112,267. Around 5.4 million reads had taxonomic signatures up to the species level (see Tables S1 and S2 in the supplemental material).

**FIG 2:**
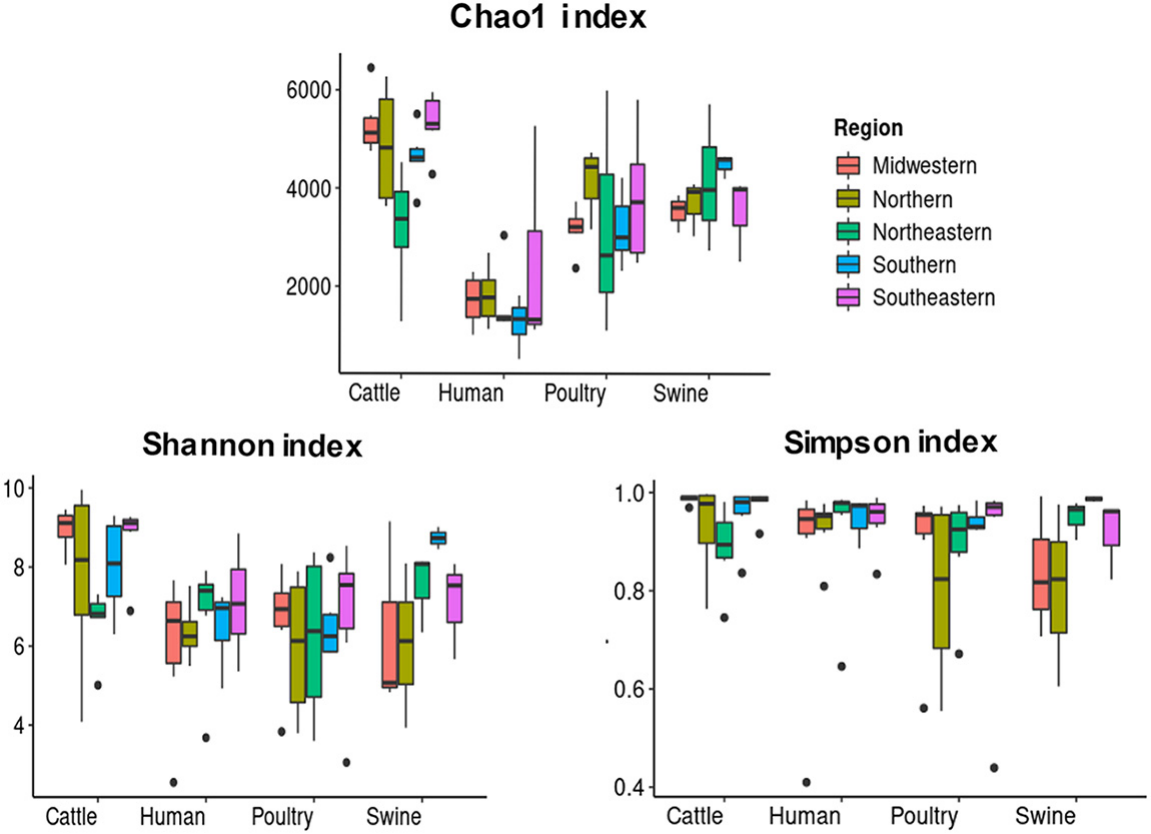
Alpha-diversity comparison of the bacterial compositions among poultry, cattle, swine, and humans, measured according to the Chao1, Shannon, and Simpson indices. Samples from five Brazilian regions are presented. Boxes represent the interquartile ranges (IQRs) between the first and third quartiles (25th and 75th percentiles, respectively), and the line inside denotes the median. Whiskers indicate the lowest and highest values within a range of 1.5-fold and the IQRs from the first and third quartiles, respectively. Dots represent outliers.

The analyzed metagenomes showed a dominance of a few microorganisms, with the 10 most abundant species accounting for nearly 20% to 50% of the total microbial diversity (Table S3). The domain *Bacteria* was overrepresented compared to other domains of life (Table S3). A total of 21,029 unique bacterial species were identified, with numbers ranging from 12,388 in humans to 16,779 in poultry (Table S3). When the diversity and richness of the human bacterial composition were compared to those of animals, the Chao1 richness estimator showed that the numbers of species were significantly higher in cattle, poultry, and swine (*P ≤ *0.05) (Fig. 2 and Table S4). When the number of distinct species present in each host was measured based on the Shannon index, the animals received similar diversity estimates, except for swine from the Southern region and cattle from the Midwestern region, which showed higher diversity (*P ≤ *0.05). Furthermore, the bacterial composition varied among cattle herds present in the Southern and Northeastern regions (Table S5). The observed diversity was not affected by the dominance of a few species, as estimated by the Simpson index, which showed elevated evenness in all hosts, with no statistically significant difference being found (Fig. 2 and Tables S4 and S5).

Interestingly, principal-coordinate analysis (PCoA) and hierarchical clustering methods showed that most human samples formed a single restricted cluster in all geographic regions analyzed, except for the Northeastern region (Fig. S1). The observation of nonclustering bacterial microbiota in cattle, poultry, and swine suggested that species were shared among these hosts (Fig. S1).

**FIG 3:**
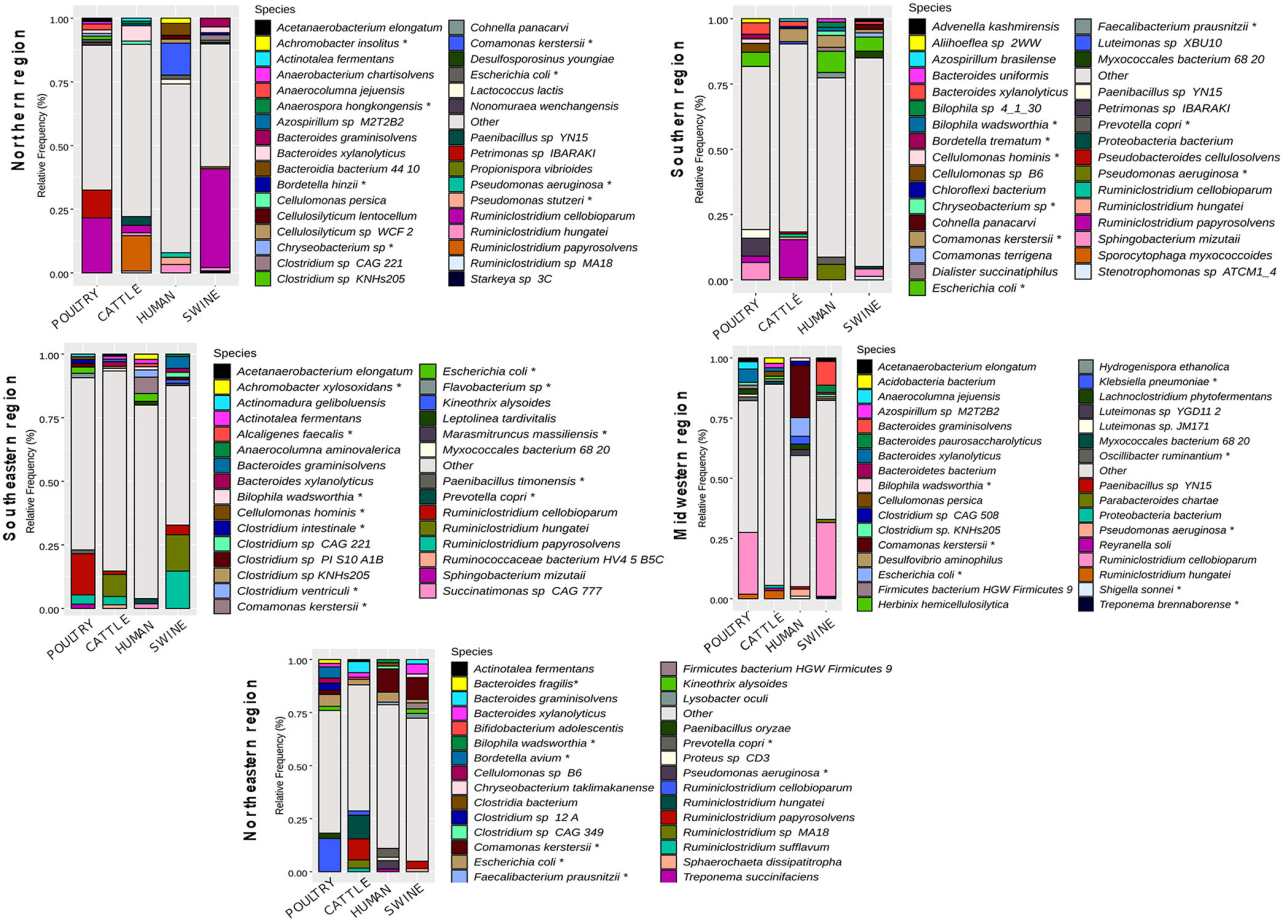
Relative frequencies of the most common species in the microbial compositions of the sampled hosts. The top 10 microorganisms from each host are presented.

The domain *Bacteria* was composed mainly of species belonging to the phyla *Firmicutes* (41%), *Proteobacteria* (29.4%), *Bacteroidetes* (14.8%), and *Actinobacteria* (8.7%) (Fig. 3 and Table S6). Cattle, poultry, and swine harbored *Firmicutes* as the most abundant phylum, represented mainly by the genera *Ruminiclostridium* and *Bacteroides*. Among these bacteria, Ruminiclostridium cellobioparum, R. papyrosolvens, and R. hungatei were the most frequent, followed by Bacteroides xylanolyticus and B. graminisolvens. Additionally, in cattle, Comamonas kerstersii (*Proteobacteria*) and Cellulomonas persica (*Actinobacteria*) were very abundant (Fig. 3). Poultry also showed considerable counts of *Petrimonas* sp. strain IBARAKI, Sphingobacterium mizutaii (both from the phylum *Bacteroidetes*), and the proteobacteria E. coli and Bordetella avium. In swine, E. coli, S. mizutaii, Bacteroides paurosaccharolyticus, and C. kerstersii were representative species. The dominance of species belonging to the *Proteobacteria* was particularly notable in humans, especially regarding the frequencies of E. coli and *C. kerstersii* (Fig. 3 and Table S6). Pseudomonas aeruginosa and Prevotella copri were also observed, as were species of the genera *Clostridium* and *Achromobacter* but at lower frequencies (Fig. 3).

### Sharing of species among humans and animals: potential scenario for cross talk colonization.

**FIG 4:**
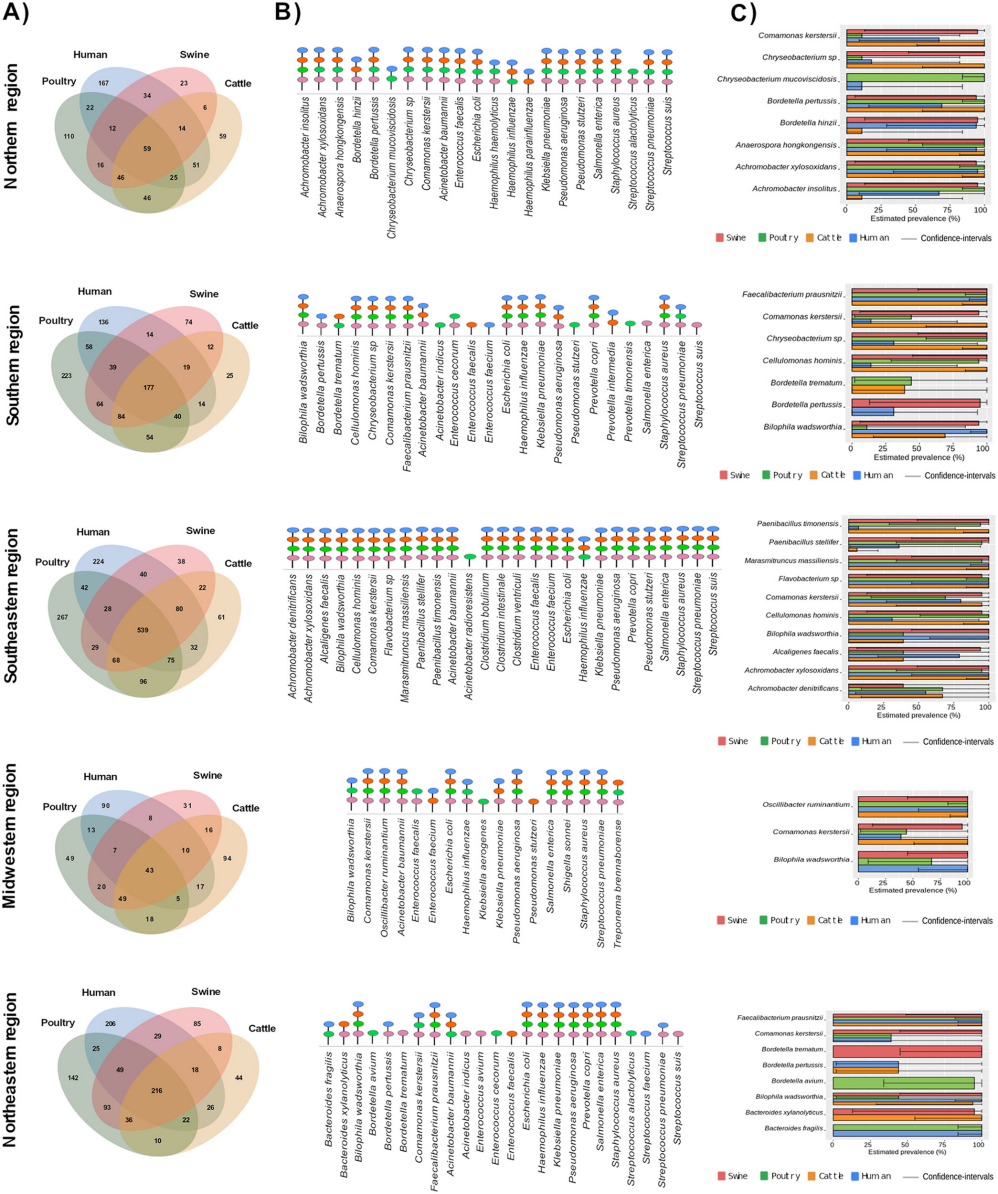
Common and exclusive species analysis among poultry, cattle, swine, and humans. (A and B) Venn diagram results for all species belonging to the 17 selected genera (comprising the 10 most abundant in host species and WHO priority groups) (A) and the predominant microorganisms from each of these genera (B). (C) Prevalence of abundant but uncommon clinical bacteria identified among the hosts within the selected genera.

The possible cross talk of bacterial colonization between humans and animals was investigated by considering the species with the highest abundances in their respective microbiomes and those described as priority bacterial groups by the WHO, thus including species of 17 distinct genera. Among the species of these genera shared among animals and humans (Fig. 4A), approximately 17 to 24 microorganisms were detected as being the most abundant in each of the Brazilian regions studied (Fig. 4B and Tables S7 and S11).

In the Northern region, *C. kerstersii* was predominant in humans (12%) (Table 1). Pseudomonas stutzeri, Achromobacter insolitus, P. aeruginosa, E. coli, and Bordetella hinzii were also present but at lower abundances than *C. kerstersii* (Table 1). More precise estimated prevalences were observed for P. stutzeri, P. aeruginosa, and E. coli (Table 1). These species were also prevalent in cattle samples (Table S7). In humans of the Southern region, E. coli (8.1%), P. aeruginosa (5.9%), and P. copri (2.7%) were abundant (Table 1); the first two species were also found in cattle, poultry, and swine (Fig. 4B and Table S8). In addition to the species described previously in hosts from other Brazilian geographic regions, the occurrence of *Clostridium* spp. (C. botulinum, C. intestinale, and C. ventriculi) was observed in both animals and humans in the Southeastern region (Fig. 4B). Surprisingly, *C. kerstersii* was more frequent in humans (23.7%) from the Midwestern region (Table 1), where Acinetobacter baumannii, Salmonella enterica, Shigella sonnei, and Streptococcus pneumoniae were also among the commonly detected species (Fig. 4B). In the Northeastern region, the species present in humans and all examined animals included E. coli and *P. copri* (Fig. 4B).

**TABLE 1:** Frequency and prevalence of predominant microorganisms belonging to 17 previously selected genera in humans from all five Brazilian geographic regions, considering species shared with animals

Region and species detected in humans	Relative frequency (%)	Prevalence (%)	Lower CI (0.95) (%)	Upper CI (0.95) (%)
Northern region				
Comamonas kerstersii	12.5479	50	19	81
Pseudomonas stutzeri	2.7876	100	61	100
Achromobacter insolitus	2.0550	50	19	81
Pseudomonas aeruginosa	1.8135	100	61	100
Escherichia coli	1.6270	100	61	100
Bordetella hinzii	1.0281	67	30	90
Klebsiella pneumoniae	0.4865	100	61	100
Acinetobacter baumannii	0.1403	100	61	100
Salmonella enterica	0.1298	100	61	100
Streptococcus pneumoniae	0.0666	100	61	100
Achromobacter xylosoxidans	0.0269	67	30	90
Anaerospora hongkongensis	0.0247	67	30	90
Haemophilus parainfluenzae	0.0200	67	30	90
Enterococcus faecalis	0.0138	83	44	99
Staphylococcus aureus	0.0084	100	61	100
Bordetella pertussis	0.0067	50	19	81
Streptococcus suis	0.0060	83	44	99
Haemophilus influenzae	0.0055	83	44	99
*Chryseobacterium mucoviscidosis*	0.0051	17	1	56
*Chryseobacterium* spp.	0.0006	17	1	56
Haemophilus haemolyticus	0.0005	33	10	70
Southern region				
Escherichia coli	8.1077	100	65	100
Pseudomonas aeruginosa	5.9414	100	65	100
Comamonas kerstersii	4.5719	14	1	51
Prevotella copri	2.7534	100	65	100
Faecalibacterium prausnitzii	2.0582	100	65	100
*Chryseobacterium* spp.	1.6796	29	8	64
Bilophila wadsworthia	1.4102	100	65	100
Klebsiella pneumoniae	1.0562	100	65	100
Acinetobacter baumannii	0.6048	100	65	100
Enterococcus faecium	0.1687	100	65	100
Salmonella enterica	0.1443	100	65	100
Streptococcus pneumoniae	0.0346	100	65	100
Staphylococcus aureus	0.0175	100	65	100
Haemophilus influenzae	0.0104	100	65	100
Bordetella pertussis	0.0046	29	8	64
Cellulomonas hominis	0.0003	14	1	51
Southeastern region				
Comamonas kerstersii	6.436	57	25	84
Clostridium botulinum	3.985	100	65	100
Escherichia coli	3.074	100	65	100
Clostridium ventriculi	2.816	57	25	84
Prevotella copri	2.085	100	65	100
*Marasmitruncus massiliensis*	2.085	86	49	99
Achromobacter xylosoxidans	2.057	71	36	92
Pseudomonas stutzeri	1.949	71	36	92
*Flavobacterium* spp.	1.848	71	36	92
Clostridium intestinale	1.507	71	36	92
Streptococcus pneumoniae	1.317	100	65	100
Bilophila wadsworthia	1.261	86	49	99
Alcaligenes faecalis	1.248	57	25	84
Pseudomonas aeruginosa	1.160	86	49	99
Staphylococcus aureus	1.143	100	65	100
Cellulomonas hominis	0.699	29	8	64
Streptococcus suis	0.604	100	65	100
Haemophilus influenzae	0.418	71	36	92
Achromobacter denitrificans	0.252	43	16	75
Acinetobacter baumannii	0.191	100	65	100
Paenibacillus stellifer	0.167	29	8	64
Klebsiella pneumoniae	0.158	100	65	100
Enterococcus faecium	0.131	100	65	100
Paenibacillus timonensis	0.118	14	1	51
Enterococcus faecalis	0.112	100	65	100
Salmonella enterica	0.082	100	65	100
Midwestern region				
Comamonas kerstersii	23.718	33	9	70
Escherichia coli	8.210	100	60	100
Bilophila wadsworthia	1.476	83	43	99
Shigella sonnei	1.135	100	60	100
Klebsiella pneumoniae	0.611	100	60	100
Acinetobacter baumannii	0.423	100	60	100
Salmonella enterica	0.140	100	60	100
Pseudomonas aeruginosa	0.093	100	60	100
Enterococcus faecium	0.053	100	60	100
Staphylococcus aureus	0.031	100	60	100
Oscillibacter ruminantium	0.017	83	43	99
Haemophilus influenzae	0.012	100	60	100
Streptococcus pneumoniae	0.007	100	60	100
Northeastern region				
Comamonas kerstersii	12.311	33	9	70
Escherichia coli	4.790	100	60	100
Prevotella copri	4.732	100	60	100
Pseudomonas aeruginosa	1.435	83	43	99
Faecalibacterium prausnitzii	1.363	100	60	100
Bilophila wadsworthia	1.083	100	60	100
Klebsiella pneumoniae	0.363	100	60	100
Bacteroides fragilis	0.313	100	60	100
Enterococcus faecium	0.111	100	60	100
Acinetobacter baumannii	0.109	100	60	100
Salmonella enterica	0.104	100	60	100
Citrobacter freundii	0.078	100	60	100
Streptococcus pneumoniae	0.023	100	60	100
Staphylococcus aureus	0.012	100	60	100
Haemophilus influenzae	0.009	83	43	99
Bordetella pertussis	0.001	33	9	70

Despite showing lower abundances, species not previously mentioned had increased prevalences in humans and some sampled animals, such as Staphylococcus aureus (from the Northern region), Faecalibacterium prausnitzii and Klebsiella pneumoniae (from the Southern region), Marasmitruncus massiliensis (from the Southeastern region), *Enterococcus* spp. (E. faecalis and E. faecium) (from the Midwestern region), and Bacteroides fragilis (from the Northeastern region) (Fig. 4C and Tables S7 and S11).

### Variability and spread of ARGs between human and livestock resistomes.

Functional analysis detected a total of 405 ARGs for 12 distinct antimicrobial classes (Table S12). Genes encoding antibiotic-modifying enzymes (*n* = 231; 57%) were the most frequent, followed by genes related to target alteration (*n* = 95; 23.5%) and efflux pump systems (*n* = 79; 19.3%).

**FIG 5:**
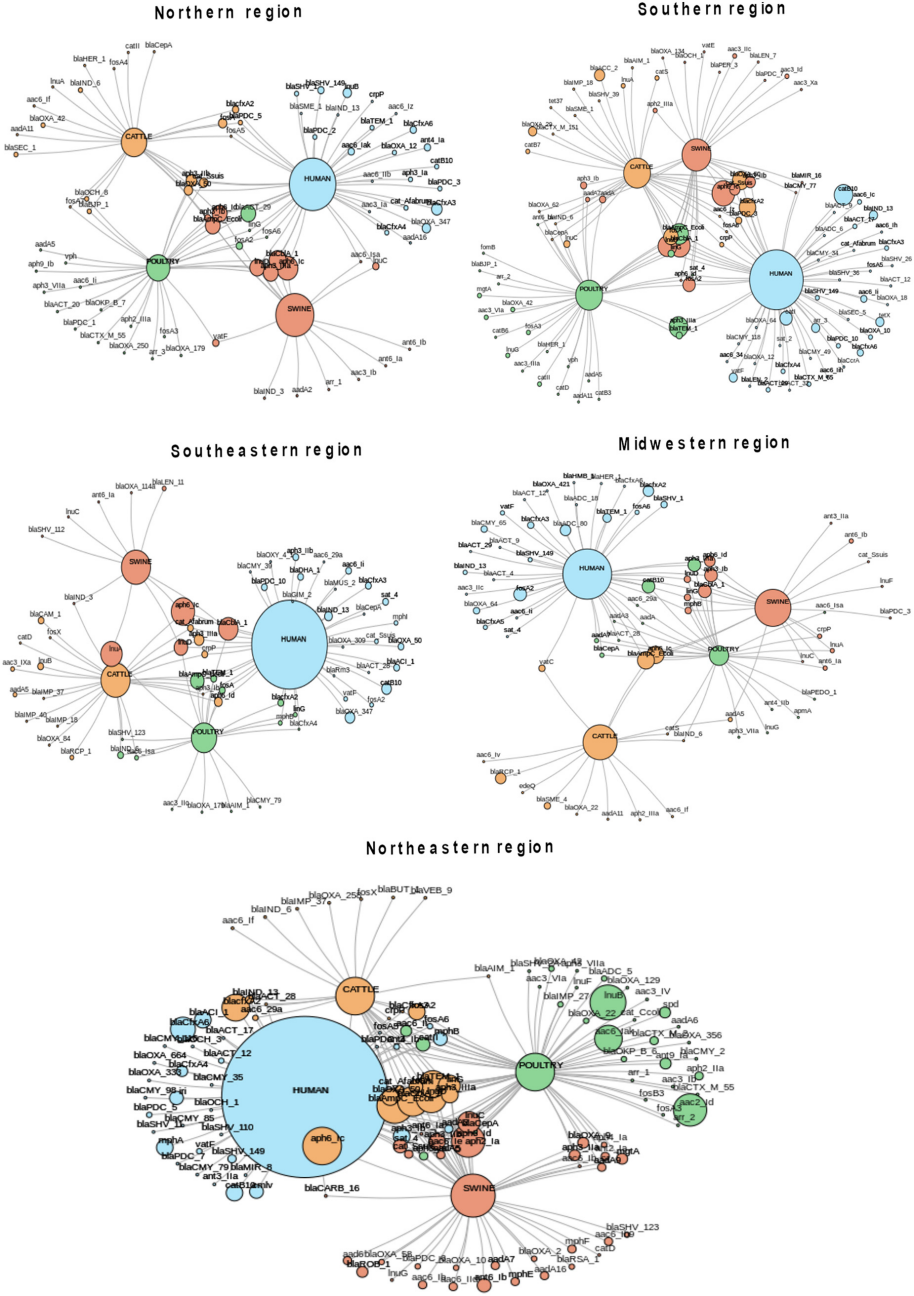
Gene network showing genes encoding products related to antibiotic inactivation according to the host and the geographic region. Circle sizes represent the abundance of each indicated ARG.

Among the genes encoding antibiotic-modifying enzymes, those encoding β-lactamases and aminoglycoside-modifying enzymes (AMEs) were the most frequent (Table S12). Intrinsic and acquired β-lactamase-encoding genes were by far the most frequent and diverse group of enzymes found (*n* = 122; 52.8%). According to Ambler’s classification, 42 β-lactamase-encoding genes belonged to molecular class C (*n* = 42; 34.4%), followed by class A (*n* = 39; 32.0%), class D (*n* = 25; 20.5%), and class B (*n* = 16; 13.1%) (Table S12). Interestingly, acquired carbapenemase-encoding genes such as *bla*_AIM-1_, *bla*_CAM-1_, *bla*_HMB-1_, *bla*_GIM-2_, and *bla*_SME-1_, which had not been previously reported in South American isolates, were observed (Table S12). The occurrence of *bla*_AIM-1_ was noted in cattle from the Southern region, in poultry from the Southeastern region, and in cattle and poultry from the Northeastern region (Fig. 5A). Similarly, *bla*_SME-1_ and *bla*_SME-4_ were found in cattle from the Southern and Midwestern regions and in humans from the Northern region (Fig. 5A). Curiously, some infrequent ARGs were identified in specific locations; for example, *bla*_GIM-2_ and *bla*_CAM-1_ were recovered from different hosts in the Southeastern region, and *bla*_HMB-1_ and *bla*_VEB-9_ were found in the Midwestern and Northeastern regions, respectively (Fig. S2). In addition, a total of 59 distinct AME-encoding genes were observed (Table S11), among which acetyltransferases (AACs) were the most frequent enzymes (*n* = 27 variants), including *aac(6′)-Ib-cr*, followed by adenylyltransferase (ANTs) (*n* = 18 variants) and phosphotransferases (APHs) (*n* = 14 variants) (Table S12).

**FIG 6:**
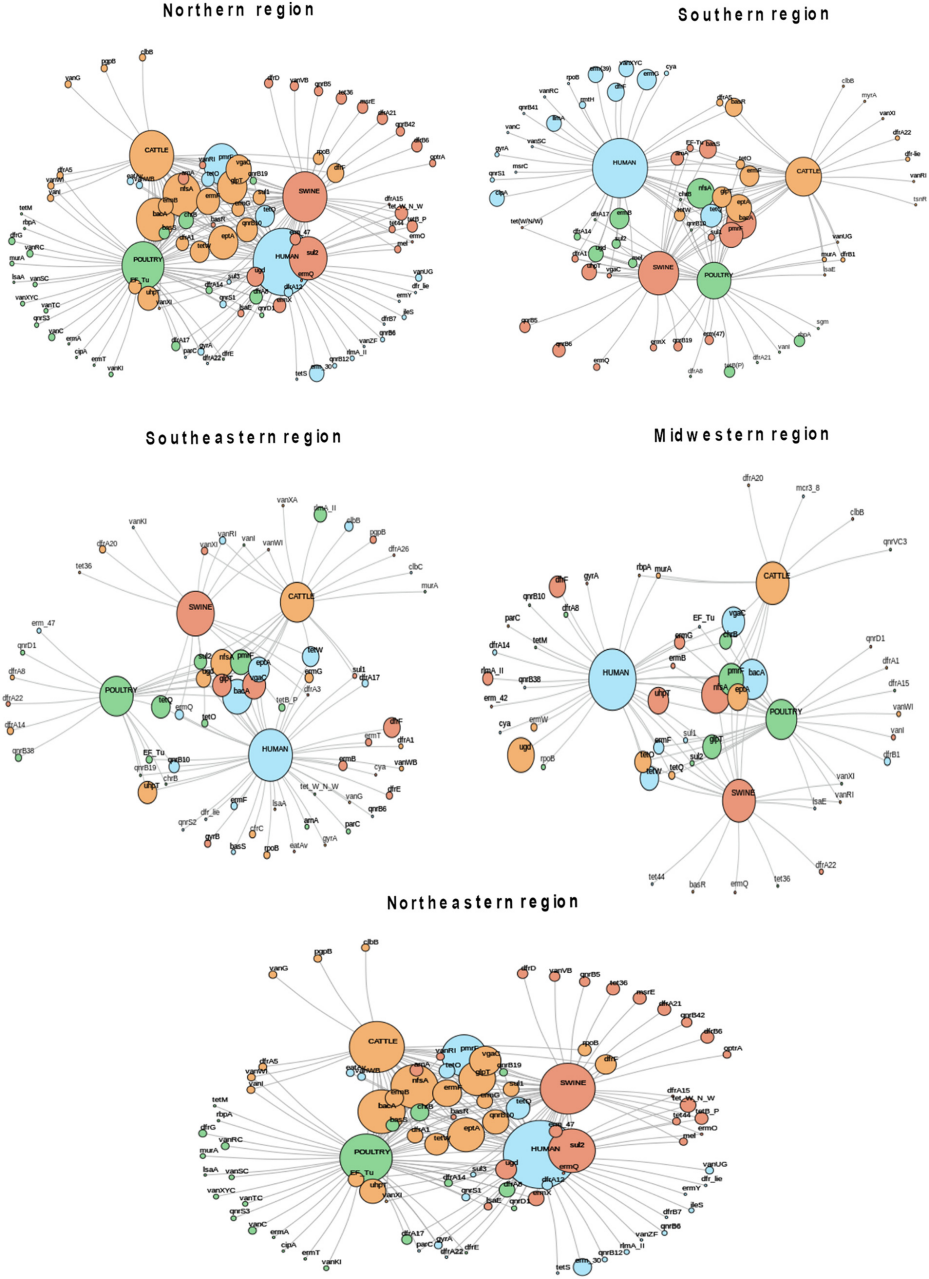
Gene network showing genes encoding products related to target alteration according to the host and the geographic region. Circle sizes represent the abundance of each indicated ARG.

In terms of target alteration mechanisms, several trimethoprim-resistant dihydrofolate reductase (DFR)-encoding genes (*n* = 22 variants) were found (Table S12), among which *dfrF* was the most frequently recorded, except in metagenomes recovered from the Northern region, where *dfrA1* and *dfrA8* were predominant (Fig. 6). In addition, Erm 23S rRNA methyltransferase-encoding genes (*n* = 14 variants), which confer resistance to macrolides, lincosamides, and streptogramins, were also commonly found (Fig. 6 and Table S12), especially *ermF* (*n* = 36), *ermB* (*n* = 27), and *ermG* (*n* = 26) in humans and poultry (Fig. 5B). Finally, nine distinct tetracycline resistance ribosomal protection protein-encoding genes (*tet* genes) were found (Fig. 6 and Table S12), among which *tetO*, *tetQ*, and *tetW* were found to be widespread in all Brazilian geographic regions (Fig. 5B). Additionally, quinolone resistance protein (Qnr)-encoding genes (*n* = 13) (Table S12) were found in all hosts, among which *qnrB10* and *qnrB19* were the most frequent variants (Fig. 5B). Interestingly, *qnrD1* was frequently found in poultry (*n* = 8), mostly from the Midwestern region, followed by humans (*n* = 4) and swine (*n* = 2) (Fig. 5B). In addition, the ciprofloxacin-modifying-enzyme-encoding gene *crpP* was found in all hosts, particularly humans, and was distributed in all geographic regions (Fig. 5B). Moreover, a total of nine variants of *fosA*, which is responsible for resistance to fosfomycin (Table S12), were also detected by the metagenome analysis and were most frequently found in humans and poultry, as were the *tet37* (cattle) and *tetX* (human) genes that confer resistance to tetracyclines and glycylcyclines (tigecycline) (Fig. 6).

Genes encoding efflux pump system components were identified, most of which belonged to the major facilitator superfamily (MFS) (*n* = 42), especially the versatile *tet* group, followed by the resistance-nodulation-cell division (RND) family (*n* = 25). (Table S12). Curiously, the frequency of multidrug and toxic compound extrusion (MATE) efflux pump systems varied according to the region evaluated (Fig. S3). These efflux pump systems were absent in swine from the Northern region, poultry from the Southern region, and, surprisingly, cattle, poultry, and swine from the Southeastern and Midwestern regions. In addition, the the small multidrug resistance (SMR) family was not found in swine from the Southeastern region (Fig. S3).

## DISCUSSION

AMR has been recognized as a serious public health concern worldwide (3, 4). The epidemiology of bacterial resistance is complex and is not restricted to humans and food-producing animals (15), as it is also associated with the environment (27) and is influenced by modern events such as international trade and travel (12, 13) and global warming (28). Because ARGs are versatile and widely distributed in different ecological niches (5, 7, 23), it is essential that AMR surveillance be based on the One Health approach (13, 17). Brazil is divided into five geographic regions, which display different sociodemographic and geographic characteristics.

In our study, cattle, poultry, and swine showed high species richness. The similar microbial structures observed in these food-producing animals, represented mainly by cellulose- and xylan-degrading *Firmicutes* species, seem to reflect their lifestyle conditions, as previously observed (2). Members of the phyla *Firmicutes*, *Bacteroidetes*, and *Actinobacteria* were found in swine and cattle, as previously reported (29, 30). In swine, *Ruminococcus* and *Bacteroides* abundance can result from early-stage animal growth, as observed previously by Han et al. (29). Jurburg et al. (31) showed that in developing chickens, the first stage was dominated by Streptococcus spp. and Escherichia spp./*Shigella* spp., which were displaced in the second stage by rapidly growing taxa, including *Ruminococcus*-like species variants (31). Beyond these taxa, distinct families and genera were identified in the animals studied in our work.

In addition to *Enterobacterales*, we observed that environmental or commensal proteobacteria, such as *C. kerstersii* (32, 33), A. insolitus (34), and B. hinzii (35), were abundant in both animals and humans and have been reported to be opportunistic agents colonizing humans. Microorganisms from distinct genera, such as P. stutzeri, Clostridium ventriculi, Faecalibacterium prausnitzii, and Bilophila wadsworthia, accounted for a proportion similar to those of some well-described antimicrobial-resistant species. As proposed previously by Woolhouse and Gowtage-Sequeria (1), small changes in animal-human interactions, such as differences in the numbers of introductions of bacteria into the host and the sizes of the susceptible populations, can lead to the spread of bacterial species, and their zoonotic potential should not be overlooked (33, 36).

In this study, our results demonstrated that resistance to β-lactams and aminoglycosides, especially that mediated by antibiotic inactivation, was the most frequent mechanism of AMR. The occurrence of antibiotic inactivation mechanisms varied slightly according to the host and geographic region and could be related to the type of feeding and handling characteristics of local livestock farming. Our results are complementary to those of previous studies (37, 38) that have evaluated environmental samples. β-Lactamase- and AME-encoding genes were also reported to be among the most frequent AMR mechanisms in water samples from Lake Bolonha, which is located in the Brazilian Amazon (37), and Brazilian mangrove regions (38).

In Brazil, the production of β-lactamases, particularly carbapenemases, by Gram-negative bacilli is the main challenge faced by physicians (39) since β-lactams have been widely used as the first line to treat serious infections (40). Interestingly, we did not observe the occurrence of *bla*_KPC_-like genes, which are the most widespread carbapenemases in *Enterobacterales* recovered from Brazilian hospitals, but a variety of class A ESBLs and class B carbapenemases were found (41). Similar results were observed previously by Alves et al. (37) in Lake Bolonha, where those authors found the presence of *bla*_IMP_-like, *bla*_VIM_-like, and *bla*_CTX-M_-like but not *bla*_KPC_-like genes. Curiously, we detected the occurrence of *bla*_AIM-1_ in livestock feces for the first time in South America, to the best of our knowledge. This class B carbapenemase-encoding gene was first described in 2012 in three P. aeruginosa clinical isolates recovered in Australia (42). To date, this type of gene has been reported only in K. pneumoniae recovered in 2019 from a patient with diarrhea in China (43). Other carbapenemase-encoding genes, *bla*_CAM-1_ and *bla*_GIM-2_-*bla*_HMB-1_, which have been described only in Canada and Germany, respectively (44–46), were also detected in our study. These findings might be justified by the detection of environmental/uncultured bacteria, which could be primary sources of these carbapenemase-encoding genes that have been further mobilized to generate resistant clinical isolates. The spread of bacterial species carrying carbapenemase-encoding genes by migratory birds around the globe to rural areas where humans and birds are in constant contact provides the opportunity for interspecies transmission and might give rise to new hypotheses (47). Recently, two studies reported the presence of endemic P. aeruginosa sequence type 277 (ST277) and A. baumannii ST79 clones carrying the carbapenemase-encoding genes *bla*_SPM-1_ and *bla*_OXA-72_ in the microbiota of migratory birds in Brazil (48, 49), respectively, reinforcing their role as hosts of MDR microorganisms.

Interestingly, we also observed the occurrence of the *crpP* gene in healthy individuals and animals for the first time in South America. *crpP* was recently described as a ciprofloxacin-modifying phosphotransferase carried by a plasmid in a P. aeruginosa strain isolated in Mexico (50). After it was initially reported in 2018, the presence of *crpP* was demonstrated in European countries (France and Switzerland) (51), Africa (Cameroon and South Africa) (52, 53), India (54), and Australia (54). It was subsequently shown that Mexican *Enterobacterales* isolates recovered in 1994 also carried this gene (55).

Our results allowed us to describe the bacterial communities and ARGs found in healthy humans and food-producing animals from distinct Brazilian geographic regions. The number of samples collected might be considered a limitation of this study; however, due to budget restrictions, we decided to obtain triplicate rectal swabs from the same host to obtain high depth and coverage of metagenomic results. In this manner, we were able to identify microorganisms to the species level and ARGs. Other authors have pointed out Brazil as a hot spot for the emergence of ARGs (16). The emergence of resistance in Brazil has a high chance of impacting all global regions because Brazil has been one of the largest exporters of food-producing animals. This study was also important for building a network that can be used in the future to initiate One Health surveillance at the national level, incorporating a higher number of centers and samples.

### Conclusion.

To the best of our knowledge, we report the first description of the bacteriome and resistome of the feces of healthy individuals and food-producing animals (poultry, cattle, and swine) collected simultaneously within the same period of time from five Brazilian geographic regions. Our results are a snapshot of the distribution of microbial species and ARGs in humans and food-producing animals. Although in most geographic regions, the microbial diversities of animals and humans were distinct, we observed a resemblance between the species isolated from humans and those from food-producing animals collected from the center located in the Northeastern region. This may suggest the influence of regional habits favoring microbiota sharing. To the best of our knowledge, we detected for the first time carbapenemase-encoding genes such as *bla*_AIM-1_, *bla*_CAM-1_, *bla*_GIM-2_, and *bla*_HMB-1_ in Latin America. In this manner, our results corroborate the importance of metagenomics as a tool for tracking the colonization of livestock and humans by antimicrobial-resistant microorganisms. Moreover, a network surveillance program named GUARANI, which was created for this study, is ready to be scaled up. It would be important to delineate the countrywide panorama of antimicrobial resistance since Brazil plays an important role in the world scenario as one of the largest exporters of meat.

## MATERIALS AND METHODS

### Ethics and regulatory approval.

Ethics approval for this study was obtained from the Research Ethics Committee (CEP) and the Committee on Ethics in the Use of Animals (CEUA) of the Universidade Federal de São Paulo (UNIFESP) (process numbers 3.116.383 and 2607170119, respectively). This project was also registered by the National System for the Management of Genetic Heritage and Associated Traditional Knowledge (process number AA1668A).

### One Health.

One Health is a collaborative, multisectoral, and transdisciplinary approach with the goal of achieving optimal health outcomes recognizing the interconnection among people, animals, plants, and their shared environment (2, 14, 18). In recent years, the One Health concept has gained importance in tackling AMR, one of the top 10 global public health threats facing humanity (56, 57).

### Sample selection.

To perform this study, rectal swabs from cattle, swine, poultry, and humans were collected between February and April 2020 from five cities located in five Brazilian geographic regions: Castanhal (Northern region) (longitude [φ] 1°17′50″S, latitude [λ] 47°55′20″W), Blumenau (Southern region) (φ 26°55′7″S, λ 49°3′58″W), Bragança Paulista (Southeastern region) (φ 22°57′8″S, λ 46°32′33″W), Dourados (Midwestern region) (φ 22°13′16″S, λ 54°48′20″W), and Fortaleza (Northeastern region) (φ 3°43′6″S, λ 38°32′36″W), as shown in Fig. 1. The GUARANI One Health Network was established based on previous research collaboration, including one researcher from each of the five distinct Brazilian geographic regions. Three rural properties of each Brazilian geographic region were randomly selected based on two criteria: (i) they should be classified as small properties according to ordinance number 8.629, 25 February 1993, established by the Brazilian Institute of Colonization and Agrarian Reform (Instituto Nacional de Colonização e Reforma Agrária [INCRA]) (https://www.gov.br/incra/pt-br/assuntos/governanca-fundiaria/modulo-fiscal), and (ii) they should raise at the same time distinct food-producing animals (cattle, swine, and poultry) for human consumption. At each property, fecal swabs were collected from cattle (*n* = 2), poultry (*n* = 2), and swine (*n* = 1). In addition, fecal swabs from two healthy adults (18 to 64 years old) who lived in urban areas served by the food produced in those small properties were also collected. In total, 107 subjects were selected for swab collection, representing cattle (*n* = 30), poultry (*n* = 30), swine (*n* = 15), and humans (*n* = 32) (see Table S1 in the supplemental material). The swabs were collected in triplicate from each subject. Briefly, Copan Amies sterile transport swabs (Copan Diagnostics, Corona, CA) were inserted 1 to 1.5 in. into the rectum and gently rotated. The same swabs were placed into the tube deep enough that the medium covered the cotton tips and were transported at room temperature to the laboratory for DNA extraction.

### DNA extraction and sequencing.

Total DNA extraction was performed using the ZymoBIOMICS DNA miniprep kit (Zymo, USA) according to the manufacturer’s guidelines. The extracted DNA was transported at 4°C to the Laboratório Nacional de Computação Científica (LNCC), where metagenomic library preparation and sequencing was performed. Libraries were constructed using the Nextera DNA Flex library preparation kit (Illumina, USA) according to the manufacturer’s recommendations. Library quality control (QC) and quantification procedures were performed using the high-sensitivity D5000 ScreenTape assay on a 4200 TapeStation system (Agilent, USA). For each sequencing run, 48 libraries were pooled by volume, and sequencing was conducted on a NextSeq 500 system using the NextSeq 500/550 high-output kit v2.5 (300 cycles) (Illumina, USA), with the system set to produce 2 × 150-bp reads.

### Bioinformatic processing and analysis. (i) Data trimming and host sequence mapping.

Raw reads were submitted to BBduk (BBMap software v.38.81 [https://github.com/BioInfoTools/BBMap]) for quality control (i.e., the identification and filtering of low-quality reads and sequencing artifacts). Reads with a quality threshold lower than a Phred score of 20 (with a sliding window of 10 bases) and a length smaller than 50 bp, Illumina adapters, and phiX174 were removed using the following parameters: minlength=50, mink=8, qout=auto, hdist=1, k=31, trimq=10, qtrim=rl, ktrim=l, minavgquality=20, and statscolumns=5. Next, the remaining reads were mapped against NCBI reference genomes for host-associated read filtering. The mappings were performed against human (Homo sapiens, GRCh38.p13 [NCBI accession number GCF_000001405.39]), poultry (Gallus gallus, GRCg6a [accession number GCF_000002315.6]), cattle (Bos taurus, ARS-UCD1.2 [accession number GCF_002263795.1]), and swine (Sus scrofa, Sscrofa11.1 [accession number GCF_000003025.6]) genomes. All mappings were done in Bowtie 2.4.156 using the end-to-end very-sensitive option (58).

### (ii) Taxonomic inference and statistical analyses.

Taxonomic analysis of the high-quality reads was performed with Kaiju software (59) (version 1.7.3) using the NR_EUK database (January 2020 version). Sequencing depth variations among samples were corrected by nonrandom library size normalization in order to make the samples comparable. For this, a factor reflecting each sample-specific library size was applied to the respective read counts [calculated as factor = (*n* trim reads *ss*/*n* trim reads *sls*) × OTU reads *ss*, where *ss* is a specific sample, *sls* is the smallest library sample size across all samples, and OTU is operational taxonomic units]. Species whose relative abundance was <0.001% were filtered to avoid false-positive results (60). Considering that a nomenclature revision has been proposed for some bacterial species, the data presented here are described according to the first name previously validated by the International Committee on Systematics of Prokaryotes. To investigate the community composition diversity, Shannon and Simpson indices and Chao1 richness estimators were computed under the relative abundance of bacterial species using the skbio.diversity.alpha_diversity function of a Python script written in the skbio package (61). The statistical significance of the diversity metrics was evaluated using analysis of variance (ANOVA) (*P < *0.05) and Tukey’s *post hoc* test on the R statistical platform. Principal-coordinate analysis (PCoA) and hierarchical clustering were conducted to determine the distances or dissimilarities between the structures of the bacterial communities. PCoA matrices were analyzed using the Bray-Curtis dissimilarity metric of the Phyloseq R package (62). Multivariate analysis of agglomerative hierarchical clustering was performed using a binary distance and the Ward.D2 method in the dendextend R package (63).

### (iii) Common and exclusive microbiota analyses.

The relationship among the microbial compositions of the different hosts in this study was determined using Jvenn viewer (64). The genera analyzed were selected based on two criteria. The first one included the genera of the 10 most abundant species from each host. The second criterion was the inclusion of 7 genera of 12 pathogens listed under distinct priority groups by the WHO: critical (Acinetobacter and Pseudomonas), high (*Enterococcus*, Staphylococcus, and Salmonella), or medium (Streptococcus and Haemophilus) (65). The order *Enterobacterales* was reported to be of critical priority by the WHO. However, genera of this order were observed to be abundant in some hosts and were previously included according to the first criterion. This resulted in 17 bacterial genera being selected. The predominant species from each genus were analyzed. To investigate the occurrence of species in an epidemiological context, an estimate of prevalence was inferred. For this, appropriate confidence intervals (CIs) were provided, accounting for the changes in variance metrics that arise from imperfect test sensitivity and specificity. The prevalence of each species was estimated using the epi.prev function in R (confidence level of 0.95, sensibility of 70%, and range of 90 to 95% specificity) and the Blaker method (66), based on cutoffs proposed in the literature (67, 68).

### (iv) *De novo* assembly and gene prediction.

To maximize the identification of ARGs in the data set, the trimmed reads of each biological replicate were grouped and assembled as a unique file sample. The assemblies of reads into contigs were performed using metaSPAdes (69) software (v.3.14) with parameter settings -k 21,33,55,77. Only contigs longer than 500 bp were included in the downstream analyses. The remaining contigs were predicted in open reading frames (ORFs) with Prodigal software (70) version 2.6.3 (applying the -g 1 -p meta options).

### (v) Identification of antimicrobial resistance genes.

Annotation and alignment against a functional database were conducted with ORFs of >50 amino acids. Predicted ORFs were aligned against the Comprehensive Antibiotic Resistance Database (CARD) (downloaded in August 2020) (71) for ARG identification. An E value of ≤1e−5, a minimum identity of 90%, and a minimum query length and subject coverage of 90% were applied as parameters. Analyses were done considering the gene assignments with the highest-scoring annotated hits. To avoid single nucleotide polymorphisms (SNPs) at specific loci within the ARGs, only genes with both 100% identity and 100% coverage of a match to a CARD reference sequence were discussed. Community detection analyses were performed to identify how groups of ARGs are clustered and can indicate interactions among the hosts. The network was constructed using the fast greedy algorithm implemented in the plot.igraph function (default parameters) available at the igraph R library (72).

### (vi) Graphics visualization.

Bar representations of microbial abundance distributions and box plots for both richness of species and AMR genes most frequently found across the hosts, also shown as heatmaps, were generated with the ggplot2 R package (73). Pairwise correlations on scatter matrices were done using the pairs function in the R language.

### Data availability.

The data sets supporting the conclusions of this article are available in the NCBI SRA (www.ncbi.nlm.nih.gov/sra) under BioProject accession number PRJNA684454.
