# Supplementary material for: Exploring the Bacteriome and Resistome of Humans and Food-Producing Animals in Brazil
Source: Microbiol Spectr. 2022 Aug 22;10(5):e00565-22. doi: 10.1128/spectrum.00565-22 (PMC9602611; doi:10.1128/spectrum.00565-22)
Supplement: Supplemental file 1 — Supplemental material. Download spectrum.00565-22-s0001.pdf, PDF file, 1.5 MB [file spectrum.00565-22-s0001.pdf]

Exploring the Bacteriome and Resistome of Humans and Food-Producing Animals in Brazil

Fabíola Marques de Carvalho[1δ], Tiago Barcelos Valiatti[2δ], Fernanda Fernandes Santos[2], Alessandro Conrado de Oliveira Silveira[3], Ana Paula C Guimarães[1], Alexandra Gerber[1], Cintya de Oliveira Souza[4], Dandara Cassu Corsi[2], Danielle Murici Brasiliense[4], Débora de Souza Collares Maia Castelo-Branco [5], Eleine Kuroki Anzai[3], Francisco Ozório Bessa Neto[2,6], Glaucia Morgana de Melo Guedes[5], Gleyce Hellen de Almeida Souza[7], Leandro Nascimento Lemos[1], Lúcio Fábio Caldas Ferraz[8], Márcia de Nazaré Miranda Bahia[4], Márcia Soares Mattos Vaz[7], Ramon Giovani Brandão da Silva[2,6], Ruanita Veiga [2], Simone Simionatto[7], Walter Aparecido Pimentel Monteiro[8], William Alencar de Oliveira Lima[4], Carlos Roberto Veiga Kiffer[9], Rodrigo Cayô[2,6], Ana Tereza Ribeiro de Vasconcelos[1γ], Ana Cristina Gales[2,9γ]*.

[1]Bioinformatics Laboratory, National Laboratory of Scientific Computing (LNCC), Rio de Janeiro - RJ, Brazil.

[2]Universidade Federal de São Paulo (UNIFESP), Laboratório Alerta, Division of Infectious Diseases, Department of Internal Medicine, Escola Paulista de Medicina (EPM), São Paulo - SP, Brazil.

[3]Regional University of Blumenau (FURB), Blumenau - SC, Brazil.

[4]Seção de Bacteriologia e Micologia, Instituto Evandro Chagas (IEC), Secretaria de Vigilância em Saúde (SVS), Ministério da Saúde, Ananindeua - PA, Brazil.

[5]Postgraduate Program in Medical Microbiology, Group of Applied Medical Microbiology, Federal University of Ceará (UFC), Fortaleza - CE, Brazil.

[6]Universidade Federal de São Paulo (UNIFESP), Laboratório de Imunologia e Bacteriologia (LIB), Setor de Biologia Molecular, Microbiologia e Imunologia, Departamento de Ciências Biológicas (DCB), Instituto de Ciências Ambientais, Químicas e Farmacêuticas (ICAQF), Diadema - SP, Brazil.

[7]Universidade Federal da Grande Dourados (UFGD), Laboratório de Pesquisa em Ciências da Saúde, Dourados - MS, Brazil.

[8]Laboratory of Molecular Biology of Microorganisms, University São Francisco (USF), Bragança Paulista - SP, Brazil.

[9]Universidade Federal de São Paulo (UNIFESP), Laboratório Especial de Microbiologia Clínica (LEMC), Division of Infectious Diseases, Department of Internal Medicine, Escola Paulista de Medicina (EPM), São Paulo - SP, Brazil.

[δ]Both authors have equally contributed to this work.

[γ]Both authors coordinated this work.

*Corresponding author.

Current Address: Laboratório ALERTA, Universidade Federal de São Paulo - UNIFESP, Rua Pedro de Toledo, 781, 6th floor, Vila Clementino, 04039-032, São Paulo - SP, Brazil. Phone/Fax.: +55 11 55715180. E-mail: ana.gales@unifesp.br.

Supplementary Figures

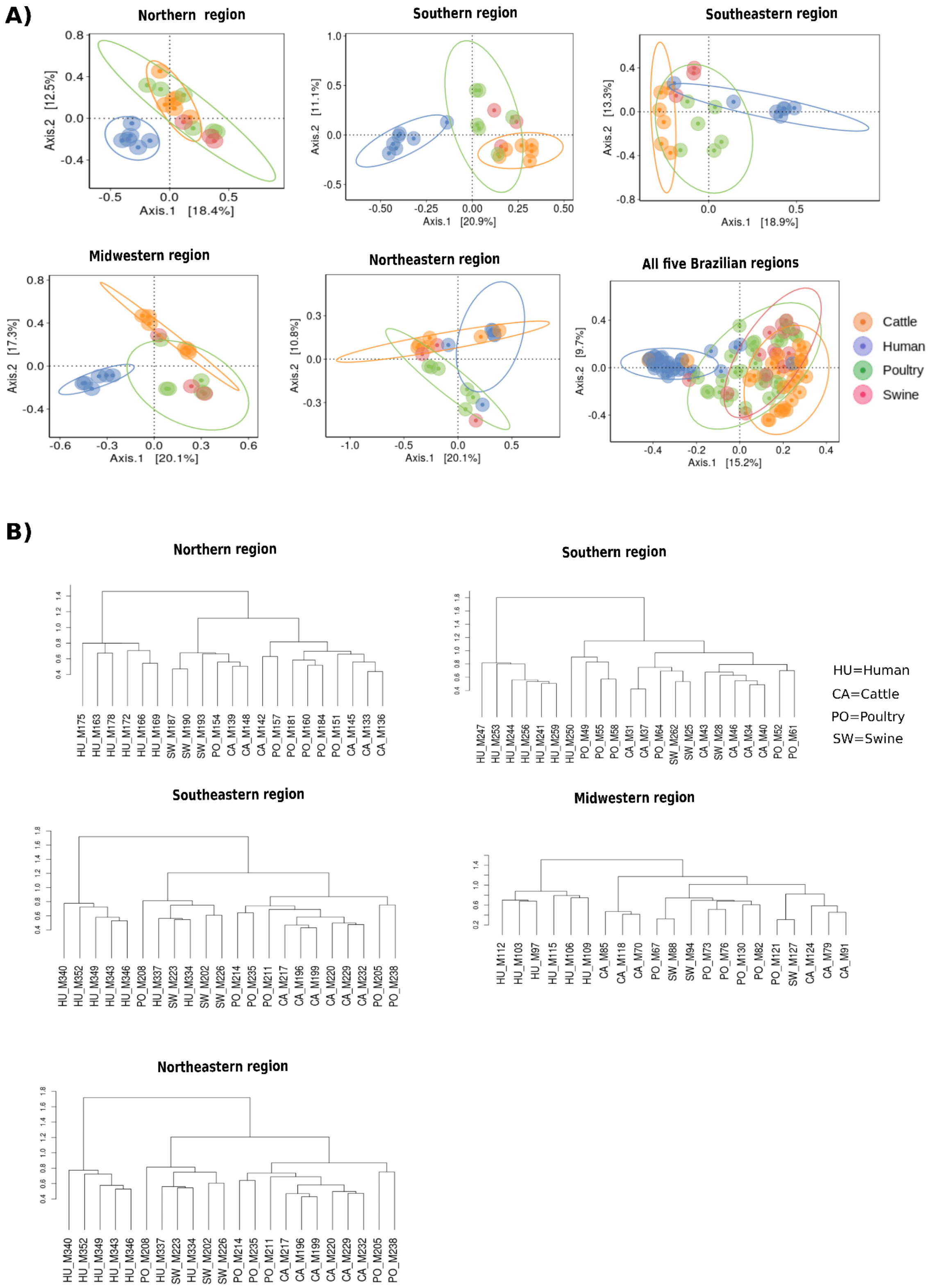

Supplementary Figure 1. Dissimilarity of the bacterial community composition indicated by principal coordinate analysis (PCoA) (A) and hierarchical clustering method (B). Samples from the five Brazilian regions are represented. (A) Each point represents a host, and the proximity or distance between points represents how different the samples are from one another. Circles represent ellipses at 95% confidence interval. The points and ellipses are colored according to the host. (B) For each region, the x-axis shows the hosts represented by HU (for human), CA (for cattle), PO (for poultry), and SW (for swine), followed by the ID sample. The y-axis shows the distance values between clusters. A clear difference in the bacterial community composition is observed for the majority of humans sampled, which was not observed in other animals.

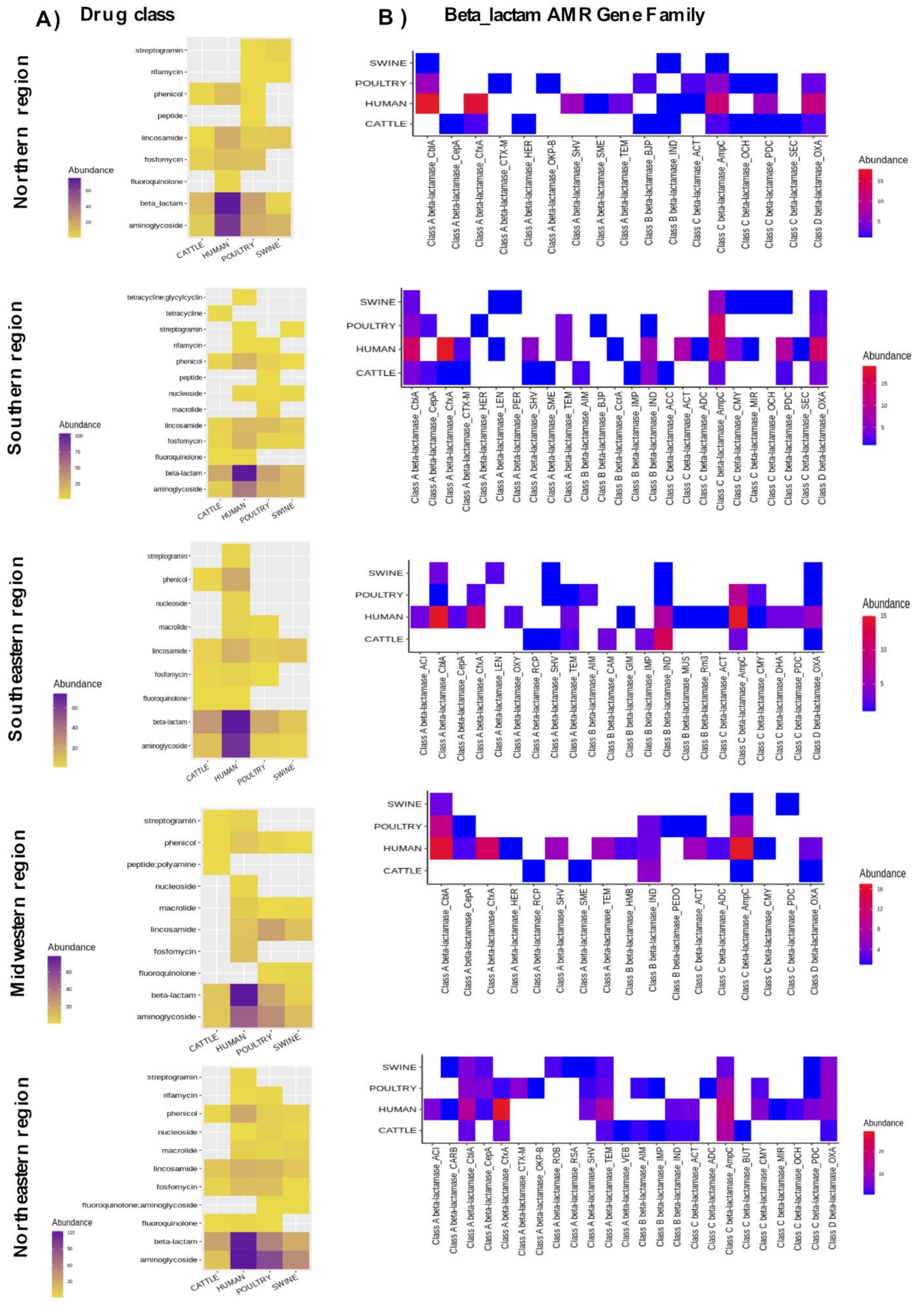

Supplementary Figure 2. Presence and abundance of antibiotic-modifying genes (A) and β-lactamase types (B) according to the host and the geographic region.

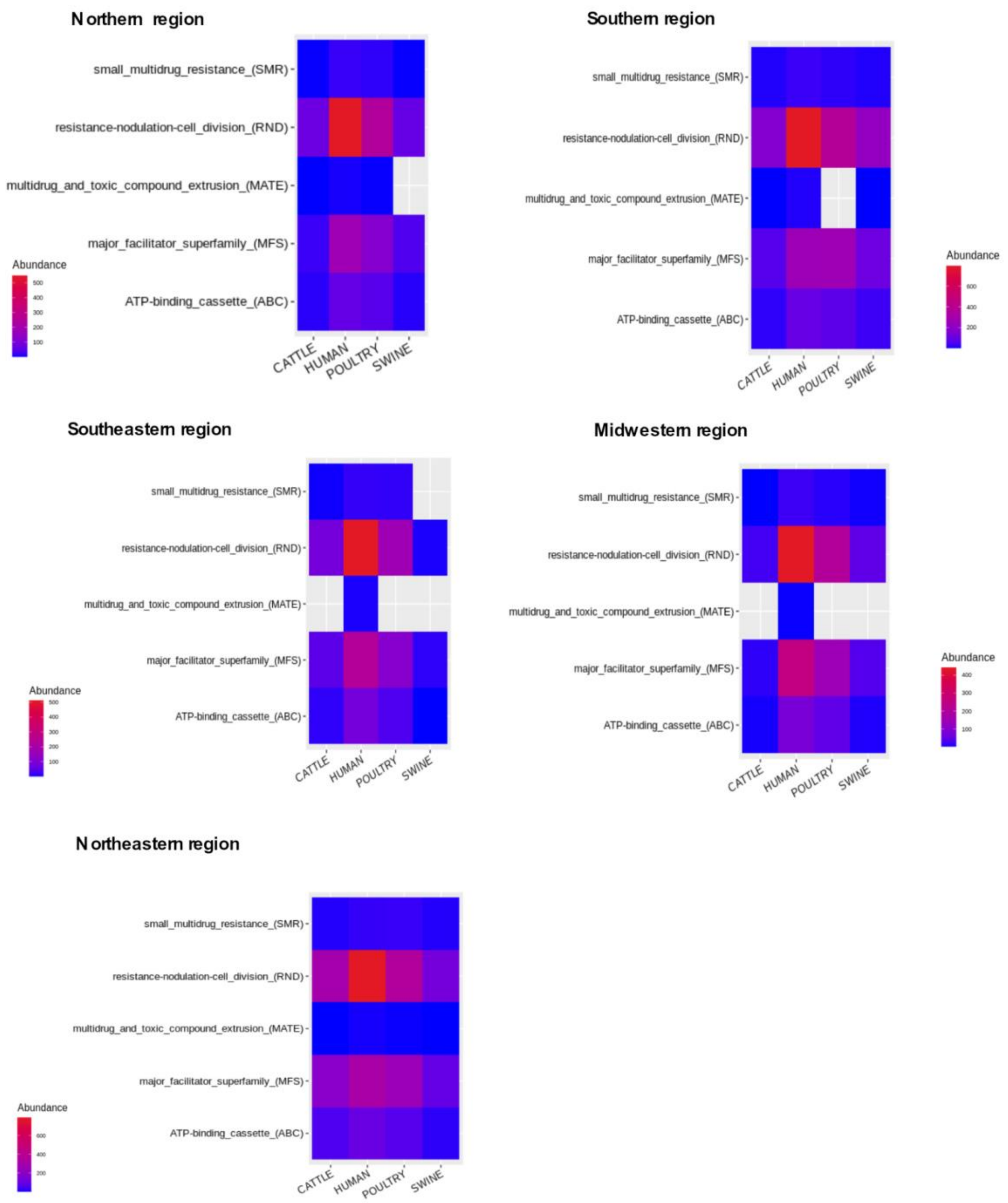

Supplementary Figure 3. Presence and abundance of the five efflux pump families according to the host and the geographic region

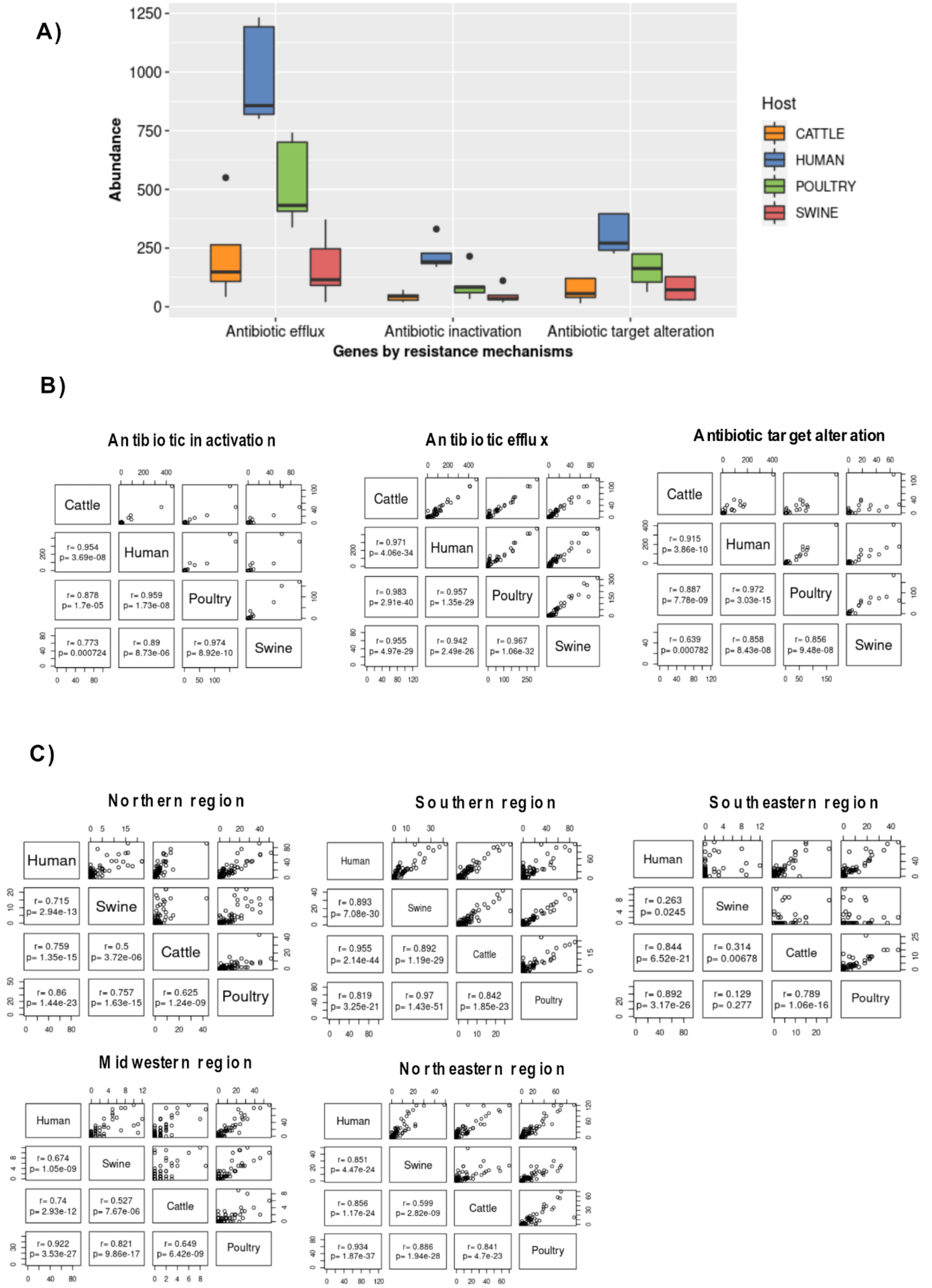
A)
Abundance
Host
CATTLE
HUMAN
POULTRY
SWINE
Antibiotic efflux
Antibiotic inactivation
Antibiotic target alteration
Genes by resistance mechanisms
B)
Antibiotic inactivation
Antibiotic efflux
Antibiotic target alteration
Cattle
Human
Poultry
Swine
C)
Northern region
Southern region
Southeastern region
Midwestern region
Northeastern region

Supplementary Figure 4. (A) The AMR genes distribution by host obtained for the resistance mechanisms: antibiotic inactivation, antibiotic efflux, and antibiotic target alteration. Boxes represent the interquartile ranges between the first and third quartiles (25th and 75th percentiles, respectively), and the line inside denotes the median. Whiskers indicate the lowest and highest values within a range of 1.5-fold and the interquartile range (IQR) from the first and third quartiles, respectively. Dots represent outliers. Scatter matrices plots showing a pairwise correlation among poultry, cattle, swine, and humans by resistance mechanisms (B) and sampled regions (C). Dots represent drug classes identified for each resistance mechanism.
